# Microwave ablation *versus* radiofrequency ablation for the treatment of pulmonary tumors

**DOI:** 10.18632/oncotarget.22308

**Published:** 2017-11-07

**Authors:** Feng Shi, Guangxiao Li, Zejian Zhou, Rongde Xu, Weike Li, Wenhang Zhuang, Zide Chen, Xiaoming Chen

**Affiliations:** ^1^ Department of Interventional Radiology, Guangdong General Hospital, Guangdong Academy of Medical Sciences, Guangzhou, Guangdong, PR China; ^2^ Department of Tumor Internal Medicine, Hexian Memorial Hospital, Guangzhou, Guangdong, PR China

**Keywords:** radiofrequency ablation, microwave ablation, pulmonary tumours

## Abstract

To retrospectively compare the efficacy and safety of radiofrequency ablation (RFA) and microwave ablation (MWA) in the treatment of pulmonary tumors, a total of 75 patients with lung tumor who underwent thermal ablation therapy in Guangdong General Hospital into the study from March 2007 to December 2014 were enrolled. Of the patients, 43 received radiofrequency ablation and 32 received microwaves ablation. The response rates, overall survival (OS), and complications rates between the RFA group and MWA group were compared. There were no significant differences in the baseline characteristics between two groups. The overall response rates of in RFA and MWA groups were 79% (34/43) and 69% (22/32), respectively, and there was no statistically significant difference between two groups (*P* = 0.309). The 1-, 2-, 3-, 5-year overall survival (OS) rates in RFA group and MWA group were 77%, 55%, 42%, 34% and 75%, 44%, 40%, 27%, respectively. No significant differences were found in the OS rates between two groups (*P* = 0.653). The complication rates were 49% (21/43) in RFA group and 50% (16/32) in MWA group; there was no significant difference between two groups (*P* = 0.921). No patients died during the perioperative period. Our study shows that no significant differences exist in efficacy and safety between RFA and MWA for the treatment of pulmonary tumors, which indicates that MWA could be a substitute therapy for RFA in terms of effectiveness and safety for treating pulmonary tumors.

## INTRODUCTION

Lung cancer is the most prevalent malignancy worldwide and the leading cause of cancer-related mortality among males. There are approximately 1.8 million newly diagnosed cases of lung cancer in 2012 [1]. Besides, the lung ranks second in the most common metastatic locations of extra - thoracic tumors [2]. For patients with early stage Non-Small Cell Lung Cancer (NSCLC), surgical resection remains the first-line therapy [3]. Lung metastasis is an indicator of advanced disease, for patients with in the lung and favorable general condition, resection can improve the prognosis [4, 5]. However, it is estimated that more than 20% of patients diagnosed with early-stage NSCLC will not be suitable for surgery, due to their advanced age, poor cardiopulmonary function, or other medical comorbidities (2).

Percutaneous thermal ablation has attracted increasing attention both as the primary therapy and as the adjuvant to radiation for patients with unresectable tumors in the lung [6, 7]. Among the ablative techniques, radiofrequency ablation (RFA) is most widely used for pulmonary ablation; meanwhile, the microwave ablation (MWA) is becoming a competitor. In the lung, the energy of RFA can be effectively deposited within the tumor with the lung parenchyma protected due to the heat insulation of the surrounding air and low electric conductivity around the tumor [8, 9]. Although this can be protective for the surrounding normal parenchyma, this may also lead to high rates of local recurrence due to the limited safety margin of ablation. Different from RFA, the energy penetration of MWA is not affected by the lower electrical conductivity and permittivity of the inflated lung. Besides, the MWA has higher intratumoral temperatures, less severe heat sink effects, shorter ablation time and larger ablation zone than RFA [10]. However, to our knowledge, few clinical comparisons of the safety and effectiveness between RFA and MWA has been published. Thus, the aim of this study was to retrospectively compare the outcome of CT-guided RFA and MWA in the treatment of lung tumors.

## RESULTS

### Baseline characteristics of RFA and MWA groups

The median follow-up time were 30 months (range, 7.7 - 88.7 months) in the RFA group and 29.7 months (range, 3.9 - 91.9 months) in the MWA group. The baseline patient characteristics for the two groups are shown in Table 1. The RFA group consisted of 33 patients with primary lung cancer (21 cases of adenocarcinoma, 12 cases of squamous cell carcinoma, 2 cases of sarcocarcinoma and one case of small cell lung cancer) and 7 patients with pulmonary metastases (1 case of hepatocellular carcinoma, 3 cases of sarcoma, 1 case of colon cancer, 1 case of mediastinal yolk sac tumor, and 1 case of angiomyolipoma). In the MWA group, there were 23 patients with primary lung cancer (13 cases of adenocarcinoma, 5 cases of squamous cell carcinoma, 1 case of sarcocacinoma, 2 cases of small cell lung cancer and 2 cases of large cell cancer) and 9 patients with pulmonary metastases (5 cases of hepatocellular carcinoma, 3 cases of sarcoma and 1 case of nasopharyngeal carcinoma). There were no significant differences in age (*P* = 0.963), gender (*P* = 0.209), tumor origin (*P* = 0.340), main tumor size (*P* = 0.403), tumor number (*P* = 0.706), UICC stage (*P* = 0.753), and ablation session (*P* = 0.987) between the two groups. The mean ablation time was 8.2 minutes (range 6-15 min) in RFA group and 5.9 minutes (range 3-12 min) in MWA group.

**Table 1:** Baseline characteristics of the study patients

	Radiofrequency ablation	Microwave ablation	*P*
No. of patients	43	32	
Age, years			0.963
Median	58.4	58.2	
Range	21-89	20-80	
Sex			0.209
Men	31 (72%)	27 (84%)	
Women	12 (28%)	5 (16%)	
Tumor origin			0.340
Primary	36 (84%)	23 (72%)	
Metastasis	7 (16%)	9 (28%)	
Tumor size, mm			0.403
Mean±Standard deviation	30.0±17.5	34.6±20.2	
Treated lung tumor			0.706
Single	40 (93%)	29 (91%)	
Multiple	3 (7%)	3 (9%)	
UICC stage			0.753
I-II	31 (72%)	22 (69%)	
III	12 (28%)	10 (31%)	
Ablation session			0.987
1	35 (81%)	26 (81)	
≥2	8 (19%)	6 (19)	

### Response rates and overall survival rates

**Figure 1:**
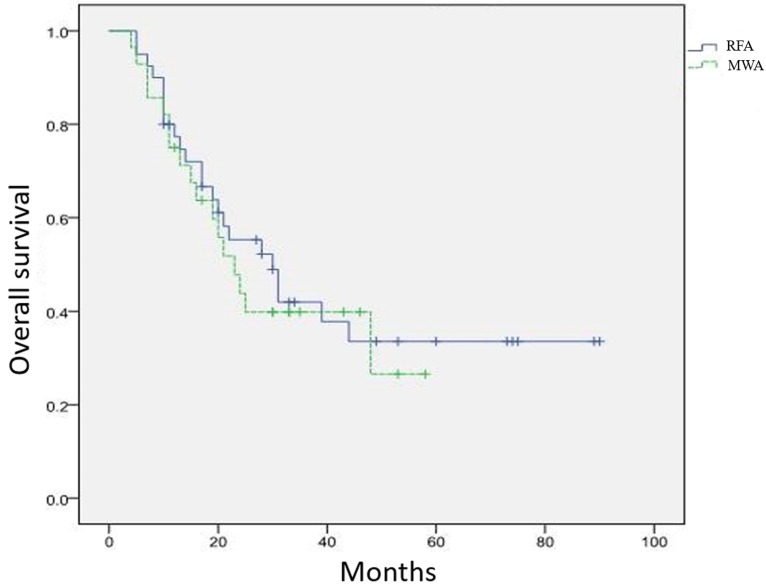
Overall survival curve in patients with pulmonary tumors who underwent RFA and MWA

All 75 patients completed the early treatment response assessment (Table 2). There were 40 patients in the RFA group and 28 patients in the MWA group receiving long-term follow-up. Among all of the patient, Complete Response (CR) was observed in 33% of patients (14 patients) in the RFA group and 31% of patients (6 patients) in the MWA group. Further, Partial Response (PR) was observed in 47% of patients (20 patients) in the RFA group and 38% of patients (16 patients) in the MWA group. The overall response rates of RFA group (79%) and MWA group (69%) were not significantly different (P = 0.309). Clinical response of the two groups is summarized in Table 2. The overall 0.5-, 1-, 2-, 3-, 5-year rates were 95%, 77%, 55%, 42%, and 34%, respectively, for the RFA group and 92%, 75%, 44%, 40%, and 27%, respectively, for the MWA group. There were no significant differences in overall survival rates between the RFA and the MWA group (*P* = 0.653) (Figure 1).

**Table 2:** Response to treatment

	Radiofrequency ablation (*n* = 43)	Microwave ablation (*n* = 32)
Overall response	34(79%)	22 (69%)
Complete response	14 (41%)	10 (31%)
Partial response	20 (47%)	12 (38%)
Stable disease	3 (7%)	6 (19%)
Progression disease	6 (14%)	4 (13%)

### Complications

There was no treatment-related mortality in both groups. In the RFA group, 21 patients experienced complications, including pneumothorax (*n* = 9), alveolar hemorrhage (*n* = 7), hemoptysis (*n* = 4), pleural effusion (*n* = 1), upper limb numbness (*n* = 1), and subcutaneous emphysema (*n* = 1). Three patients in the RFA group were diagnosed with two different complications simultaneously. In the MWA group, 21 patients experienced complications, including pneumothorax (*n* = 6), alveolar hemorrhage (*n* = 3), hemoptysis (*n* = 4), pleural effusion (*n* = 1), upper limb numbness (*n* = 1), subcutaneous emphysema (*n* = 1), and lung abscess (*n* = 1). The difference in complication rates between the two group did not reach statistical significance (*P* = 0.921).

## DISCUSSION

Percutaneous image-guided tumor ablation has been widely applied to solid tumors, including liver, kidney, bone, breast, and adrenal glands for almost two decades [11]. This technique is now proved to be an important tool in the treatment of primary and secondary lung tumors. Percutaneous thermal ablation offers patients a repeatable, effective, safe and low-cost treatment for lung malignancies either after or concurrently with radiotherapy or systemic therapy [11]. The NSCLC guideline published by national comprehensive cancer network (NCCN) recently suggested that ablation could be an option for patients with unresectable stage IA NSCLC, selected patients with multiple pulmonary lesions and those with recurrence in the lung [12]. A prospective multicenter trial [13] describing 2-year outcomes in patients with medically inoperable, stage IA NSCLC who receiving CT-guided RFA was reported by Dupuy et al. The 1-, and 2-year OS rate were 86.3% and 69.8%, respectively, and there was no significant change in the Forced Expiratory Volume (FEV1) or Diffusing capacity of the lung for carbon monoxide (D_LCO_) after RFA. There were some studies focusing on the effectiveness of ablation as a first-line therapy for early lung cancer beyond stage IA. The study of Kodama et al, consisting of 33 patients with 35 lung tumors ranged from 2.0cm to 4.4cm (mean 3.0cm), performed radiofrequency ablation (RFA) by using a multiple-electrodes witching system [14]. The 1-year overall survival rate was 81.2% and the local tumor progression rate was 12.7%. In the treatment of intermediate and advanced stage NSCLC, RFA is not only used as a combined therapy with chemotherapy and radiotherapy, but also as a salvage therapy after conventional treatment. Cheng et al. performed percutaneous thermal ablation as salvage therapy for recurrent NSCLC after radiotherapy [15]. The median progression free survival time was 14 months, and median OS time was 35 months. Besides, percutaneous thermal ablation has been widely used in the treatment of lung metastases. De Baère et al. performed 642 RFA in the 1037 lung metastases for 566 patients with primary tumor of the colon (34%), rectum (18%), kidney (12%), soft tissue (9%) and miscellaneous (27%); the median OS time was 62 months and 4-year local progression free survival rate was 89% [16].

Among these percutaneous thermal ablations, the effectiveness of RFA has been well documented, but little is known about MWA. Liu H et al [17] used MWA to ablate 16 lung lesions (mean 2.4cm) in 15 patients with stage I NSCLC, with an average of 2.1 sessions per patient. The median follow-up time was one year, yielding 9 cases of CR and 7 cases of PR. The study found that the diameters of all the progressed tumors were larger than 3cm. MWA uses high-frequency electromagnetic waves (300 MHz to 300 GHz) to engender tissue heating effects; while RFA utilizes the heat generated from the medium frequency alternating current. Due to their difference in mechanism, MWA owes a much larger zone of active heating than RFA.

Recent studies have been focused on the comparison of RFA and MWA in the treatment of liver cancer [18], but the effectiveness of theses two therapy in the management of lung cancer remains unclear. Brace et al. performed a study using a normal porcine lung model to compare RFA and MWA with equivalently sized applicators, which showed that MWA creates larger and more circular ablation zone than RFA [19]. Zemlyak et al [20] retrospectively compared the effectiveness of resection (*n* = 25), RFA (*n* = 12) and MWA (*n* = 27) for patients with stage I NSCLC; their study revealed that the treatments efficacy were equivalent among the three groups (3-year OS rates of 87.1%, 87.5%, and 77%, respectively; cancer specificity OS rates of 87.1%, 87.5%, and 77%, respectively; DFS rates of 50%, 90.2%, 45.6%) . Our study suggested there was no statistical difference between RFA and MWA in tumor local control rate and survival. This expands upon initial studies demonstrating equitable OS between the two groups and adds to the body of work supporting the use of RFA and MWA as equally effective treatments for lung tumors.

As for complications, a study [21] compared between the two procedures using rabbit model. They had 10 rabbits in each group, and the cases of procedure-related death, pneumothorax, abscess and chest wall burns were 1, 4, 1, and 0, respectively, after RFA, and 0, 4, 1, and 4, respectively, after MWA. The results showed no significant difference. During a pathological examination, they found that necrosis, edema and peripheral lymphocytes infiltration were identified in each rabbit, and there was no significant difference between the two groups from day one to day three (*P* = 0.17). Therefore, the study suggested that RFA and MWA achieved equal safety in the lung ablation. Carrafiello et al [22] reported the complications of 45 patients with lung cancer after RFA (29 patients with 36 lesions) and MWA (16 patients with 17 lesions), which demonstrated that the most common complications were pneumothorax, pleural effusion, and subcutaneous emphysema, successively. They believed that both techniques were safe. As for our study, there was no procedure-related death. Complications were observed in 21(48.83%) patients after RFA and 16(50%) patients after MWA. No significant difference was found between the two groups.

Our study has three main limitations. First, our study is a retrospective study with a nonrandomized design; thus, the introduction of selection bias is unavoidable. Second, the sample size in our study is relatively small. Multicenter large sample size randomized trials should be carried out in the future to further test the curative efficiency of RFA and MWA for lung tumors. Third, our data were analyzed without taking into account the radiotherapy or systemic therapy before or after thermal ablation that may impact on OS.

In conclusion, these preliminary studies showed there is no significant difference in the effectiveness and safety between MWA and RFA in treating lung tumors. MWA could be considered an alternative technique for RFA in treating lung tumors.

## MATERIALS AND METHODS

In this retrospective cohort study, we reviewed the records of 75 consecutive patients who underwent RFA or MWA for lung neoplasms, from a database that was collected at the Guangdong General Hospital from March 2007 to December 2014. The indications for lung tumor ablation were that the number of tumors in the lung is fewer than four and the size of the largest tumor is less than 5 cm in diameter. All the lung malignancies were pathologically confirmed by biopsy. The diameter of the tumors was measured using computed tomography (CT). Our study protocol was approved by the institutional review board and informed consent was obtained from all patients. The inclusion criteria included: (a) The patient was diagnosed with malignant neoplasms of the lung. (b) The patient was considered nonsurgical candidates medically or the patient refused surgery. *(c)* The blood oxygen saturation level of the patient was 92% or higher with room air. *(d)* The Eastern Cooperative Oncology Group performance status was 0 or 1. *(e)* Patient life expectancy was greater than 2 months. Exclusion criteria were as follows: (a) patients with severe coagulopathy (b) patients were not able to give written consent. The final study group comprised 43 patients in the RFA group and 32 patients in the MWA group. The patient characteristics are shown in Table 1.

### Radiofrequency ablation

**Figure 2:**
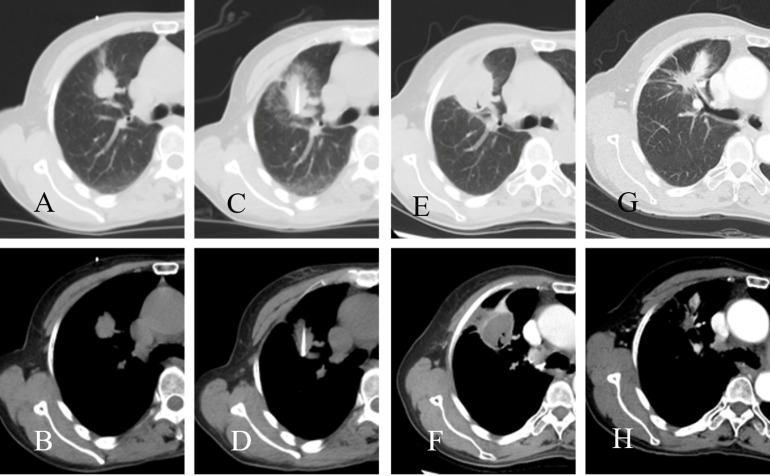
CT images of a patient who underwent radiofrequency ablation **A.**, **B.** before radiofrequency ablation; **C.**, **D.** during radiofrequency ablation **E.**, **F.**; three months after radiofrequency ablation; **G.**, **H.** 24 months after radiofrequency ablation.

RFA was performed with a commercially available system (RITA1500 Medical Systems, Mountain View, Calif) to generate up to 250 W of energy. A multitined expandable needle electrode (StarBurat Xli) was used for tumors more than 3 cm in the longest dimension, while a straight cooled tip electrode (UniBlate) was used for tumor ≤3cm. Two dispersive electrode-grounding pads were positioned on the patients’ thighs. The RFA was performed under moderate sedation and 1% lidocaine local anesthesia was given intradermally. The patients were positioned in prone, supine or lateral position on the CT (Lightspeed16; GE Healthcare, Waukesha, WI) scan bed based on the location of the tumor. The electrode was inserted into the tumor under CT guidance at a planned angle and depth (Figure 2). Power settings and ablation times were determined in accordance with standard recommendations by the manufacturer. Overlapping techniques were applied for tumors larger than 3 cm for complete ablation of the entire tumor. All tumors located in one lung were expected to be treated during a single session.

### Microwave ablation

**Figure 3:**
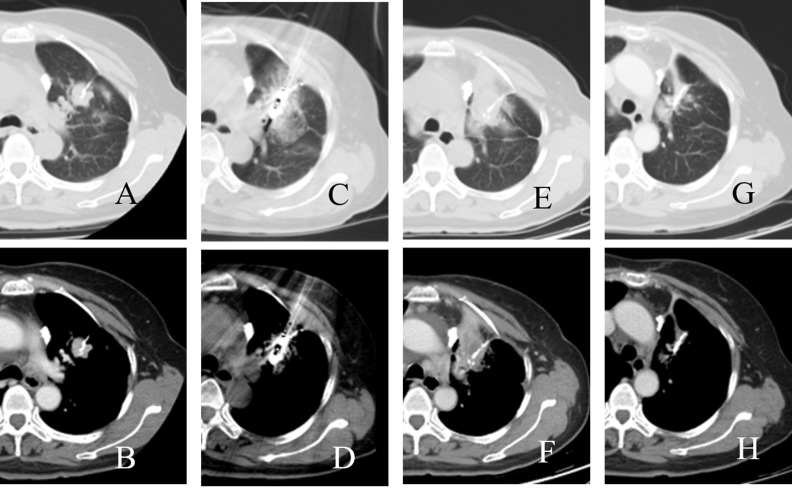
CT images of a patient who underwent microwave ablation **A.**, **B.** before microwave ablation; **C.**, **D.** during microwave ablation **E.**, **F.**; three months after microwave ablation; **G.**, **H.** 54 months after microwave ablation.

All procedures were performed with patients under moderate sedation and CT (Lightspeed16; GE Healthcare, Waukesha, WI) guidance. The microwave ablation therapeutic instrument (KY-2000, Yigao, Nanjing, China) we used could produce 0 to150W of power at a frequency of 2450 MHz. After local anesthesia, a microwave antenna (14G outside diameter, using the water circulation cooling system) was placed into the lesion (Figure 3). For tumor no more than 3 cm in maximum diameter, one antenna was performed, while two antennae were used for tumor greater than 3 cm. Ablation was performed with a power of 60 to 80 W for 4 to 8 minutes per site. Based on the tumor size and shape, antennae were placed sequentially at 1 to 8 different sites in the tumor.

### Follow-up

Follow-up contrast-enhanced CT examination or PET/CT was scheduled at three and six months after RFA treatment, and at 6-month intervals after that. All follow-up images were reviewed and evaluated independently by two senior radiologists. The tumor size, the presence of contrast enhancement, necrosis, and any metastasis were recorded. The potential complications such as pneumothorax, alveolar hemorrhage, subcutaneous emphysema pleural effusion, and lung abscess were monitored and reported if present. To assess early treatment response, we used the modified Response Evaluation Criteria in Solid Tumors (RECIST) criteria [23] which came up from University of Pittsburgh Medical Center (UPMC) at the ablated sites based on imaging review of 3-6 month after ablation. Complete Response (CR) was defined as any two of the following: (a) Lesion disappeared or reduction to 25% or less of the original size; (b) formation of the sac in the lesion; (c) SUV<2.5 in PET imaging. Partial response (PR) was defined as any of the following: (a) a reduction of largest diameter of 30% or more; (b) the formation of a central liquid sac or central necrosis; (c) a decreased SUV or a decreased area of FDG uptake. The stable lesion was defined as any of the following: (a) the reduction of largest diameter of less than 30%; (b) a solid mass appearance of the lesion, without central necrosis or cavity formation;(c) unchanged SUV or unchanged area of FDG uptake. Progression was defined as any 2 of the following: (a) an increase of the largest diameter of 20% or more; (b) a solid mass appearance and invasion of adjacent structures;(c) Higher SUV. Overall survival rates were estimated by the Kaplan-Meier.

### Statistical analysis

Patient characteristics were compared between the RFA and MWA groups. Differences in categorical variables were tested by Chi-squared test. Fisher exact test was performed when the number of cases was less than 5 or overall frequency was less than 40. The CR, PR, stable lesion, and the progression of the two groups were analyzed using the Kruskal-Wallis H-test. OS rates were estimated using the life-table method. Survival curves of the two groups were estimated and compared using the Kaplan-Meier analyses. Covariates, such as age, gender, tumor origin, tumor size were included in the analysis. All analyses were performed with SPSS 19.0 for windows. All differences were tested with a level of significance of 0.05.
